# Morphologic Differentiation of the Exotic Parasitoid *Eupelmus pulchriceps* (Hymenoptera: Eupelmidae) in the Galapagos Archipelago

**DOI:** 10.1007/s13744-023-01097-3

**Published:** 2023-12-22

**Authors:** Nicolas David Camargo-Martinez, Mariana Camacho-Erazo, Angela R. Amarillo-Suárez, Henri W. Herrera, Carlos E. Sarmiento

**Affiliations:** 1 Lab de Sistemática y Biología Comparada de Insectos, Instituto de Ciencias Naturales, Univ Nacional de Colombia, Bogotá, Colombia; 2 Museo de Entomología, Facultad de Recursos Naturales, Escuela Superior Politécnica del Chimborazo, Riobamba, Ecuador; 3 Depto de Ecología y Territorio, Facultad de Estudios Ambientales y Rurales, Pontificia Univ Javeriana, Bogotá, Colombia

**Keywords:** Morphological differentiation, Allometry, Biological invasions, Parasitoid

## Abstract

The historical and geographical properties of the archipelagos allow a detailed study of species diversification, and phenotypic traits can indicate the extent of such processes. *Eupelmus pulchriceps* (Cameron, 1904) is an exotic species to the Galapagos archipelago, and generalist parasitoid that attacks a beetle species that consumes the seeds of the invasive shrub *Leucaena leucocephala* (Lam.) de Wit*.* Despite extensive sampling, the wasp is recorded only in Santa Cruz and San Cristobal islands of the Galapagos archipelago. Thus, using 112 female wasps, we compare body size, proportion, and allometric differentiations within and between the two islands. There were no body size differences between islands. A PerMANOVA indicates differences between the islands and a single differentiation between two localities of one island. Allometric differences between islands were not the same for all structures. These results are consistent with the greater distance between islands than between localities and suggest a differentiation process. The variables with allometric differentiation are associated with wings and ovipositor, possibly responding to different ecological pressures. It is interesting that this parasitoid, recently arrived at the archipelago, is already showing differentiation. Also, it is essential to monitor the behavior of these wasps in the archipelago, given their potential to access other species affecting the trophic interactions of the local biota.

**Supplementary Information:**

The online version contains supplementary material available at 10.1007/s13744-023-01097-3.

## Introduction

The history and geographical characteristics of the archipelagos allow a detailed study of speciation since islands provide multiple opportunities for the isolation of small populations (Sequeira et al. 2000), as each island represents a replicated natural experiment and thus offers excellent statistical power, high potential for comparative studies, and a good setting for testing ideas about species diversification (Parent et al. 2008; Arnedo and Hormiga 2020). Thus, genotypic and phenotypic differences of a single species occurring on different islands or habitats within an island may suggest ongoing speciation (Pfingstl and Baumann 2017). Studies have focused on identifying genotypic differences, but as the phenotype is a major subject of selection, it is necessary to characterize the differences in phenotypic traits to determine to what extent these attributes contribute to the survival and reproduction of foreign populations and their potential for adaptation (Colautti and Lau 2015). Morphologic variation can be due to genetic or environmental changes and usually the latter have been considered as a random event without impact in evolution; however, phenotypic plasticity has recently gained acknowledgement as a potential source for differentiation of lineages, and thus, documenting small variation in populations that have arrived to novel environments is valuable to understand the fate of these populations in an evolutionary context (West-Eberhard 2005; Pfenning et al. 2010; Casasa and Moczek 2019); in particular, changes in body proportions and body size could suggest differential selective pressures and investment trends (Citeli et al. 2022; Daskin et al. 2023; Stern and Emlen 1999; Viscosi et al. 2012; Casasa and Moczek 2019).

However, the expected relationship between population differentiation and island characteristics is not always fulfilled due to multiple factors such as dispersal capacities of organisms, the timing of arrival to the island (Parent et al. 2008; Pfingstl and Baumann 2017; Colautti and Lau 2015), multiple dispersal routes, or in situ differentiation events (Lomolino et al. 2017). The early morphological differences in both size and proportions of relevant functional structures between populations of recently arrived species to archipelagos are interesting as they may show early stages of speciation and contemporary evolution (Colautti and Lau 2015).

The Galapagos archipelago is an excellent location for such studies, and because of that, it has occupied a unique position in the history of evolutionary studies, continuously shaping our understanding of evolutionary biology (Parent et al. 2008; Arnedo and Hormiga 2020; Husemann et al. 2015). The archipelago is an excellent location because (1) the islands are of oceanic origin which means they never have been in contact with the continent, (2) have different sizes and distances between them and to the mainland, and most recently, and (3) have suffered multiple processes of human intervention such as introduction of exotic species, increasing urbanization, and tourism activities (Causton et al. 2006). All these factors have been demonstrated to cause rapid differentiation among populations. It is estimated that more than 500 species of insects have been introduced to the Archipelago (Bulgarella et al. 2022).

*Eupelmus pulchriceps* (Cameron, 1904) (Hymenoptera: Eupelmidae) is a generalist species of wasp (Pérez Benavides et al. 2020), it was described in 1908 from Nicaragua and introduced in 1934 to Hawaii from Guatemala populations as a biocontrol agent for the weevil *Anthonomus eugenii* Cano (Curculionidae) (Gibson 2011). Worldwide *E. pulchriceps* is reported as a parasitoid of 76 insect species from 21 families distributed in six orders (Pérez Benavides et al. 2020) and is widely distributed in the Nearctic and Neotropical regions (Estrada Virgen et al. 2019). Despite an extensive structured sampling of Hymenoptera in the thirteen major islands of the archipelago conducted along three years using several sampling methods that significantly increased the number of species (Picón-Rentería et al. submitted), *E. pulchriceps* is recorded only in Santa Cruz and San Cristobal islands which are the more populated of the archipelago (Amarillo-Suárez et al. submitted). These data suggest a relatively recent arrival of the species to the archipelago.

This species, like many Eupelmids, exhibits interesting mesosoma modifications to enhance a powerful kick from the mid-legs, turning the flights into wing-assisted jumps as the main displacement mechanism (Gibson 1986, 1995). This poor flight ability may also have influenced its polyphagy, larval voracity, and hyperparasitic behaviors (Gibson 1995). Another interesting characteristic to explore, indicated by Gibson (2011), is that the species exhibits changes in the length of the ovipositor valves unassociated with body size. This morphometric behavior may facilitate host changes as it has also been proposed for Ichneumonoidea (Eggs et al. 2018).

Due to its exotic condition for the Galapagos islands and its generalist biology, *E. pulchriceps* is an interesting model to analyze the degree of morphological and persistent differentiation in the context of founding populations in an isolated archipelago. Thus, by analyzing variation in size and proportions between populations, this study aimed to identify if there is morphological differentiation, especially for structures important for their mobility and parasitoidism, between populations of *E. pulchriceps* between and within islands comparing localities.

## Materials and methods

### Morphological characterization

**Fig. 1:**
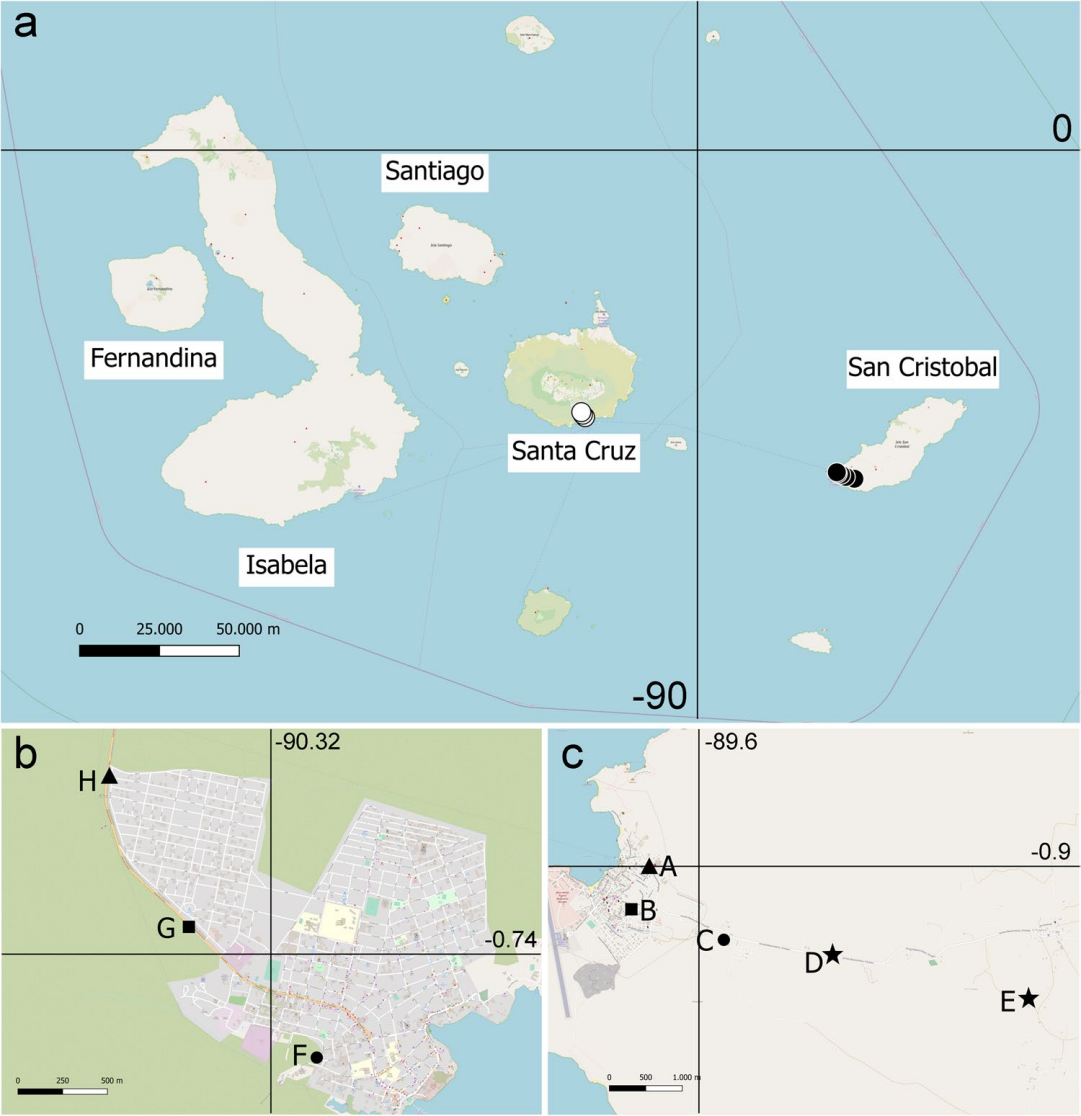
Distribution of the sampling areas for *Eupelmus pulchriceps* in the Galapagos islands (**a**), Santa Cruz (**b**), and San Cristobal (**c**). Locations of the same shape belong to the same group for analyses. Locations D and E from San Cristobal Island were excluded from within island analyses due to small sampling size

The samples came from a study of seed predators of nine legume plants conducted between 2018 and 2021 from Santa Cruz (2018), San Cristobal (2019 and 2021), Floreana (2019), and Isabela (2019) islands where a total of 105 trees and 550 kg of seeds were screened for the seed predator beetle *Acanthoscelides* sp. (Chrysomelidae, Bruchinae) and its hymenopteran parasitoids (Amarillo et al. submitted). No wasps collected from 2021 from San Cristobal were included in the analyses. Specimens of *E. pulchriceps* were obtained from *Leucaena leucocephala* (Lam.) de Wit. seeds collected at three sampling locations from Santa Cruz Island separated on the average 1.3 km (range 0.94–1.9 km) (cool season August–September) and five localities from San Cristobal Island (end of the hot season, May). In San Cristobal, due to sample size, only three locations were included in the analysis within the island. These three locations are separated on average by 1.1 km (range 0.5–1.4 km) apart. The two islands are separated by 83 km (Fig. 1). No records of the wasp nor the host beetle came from Floreana or Isabela. Of the 299 females collected, a random subsample of 40 females from Santa Cruz and 72 from San Cristobal was used for the analyses.

Seed pods of the trees were collected and stored in hermetic plastic bags and brought to laboratories at the Charles Darwin Foundation in Santa Cruz Island, and to the Directorate of the Galapagos National Park in San Cristobal Island. Both locations were rooms that experience similar weather conditions of the area as no air conditioning were provided (Santa Cruz mean temperature 22.5 °C, range 19.0–30.5 °C, humidity 87–98%, San Cristobal mean temperature 26 °C, range 20.0–31.8 °C, humidity 87–96%) (Climatology Database of the Charles Darwin Foundation n.d.). Once there, all seed pods were open, and seeds were placed in plastic receptacles recording collection date and location. The receptacles were inspected twice daily, and emerging insects from the seeds were stored in alcohol vials with the location ID. The receptacles were inspected until no insects emerged from the seeds for seven consecutive days. The longest waiting time for emergence of individuals was 20 days. This ensured that all insects surviving and completing development in the seeds were collected. The *L. leucocephala* seeds provided only three species: the parasitoid wasp *Eupelmus pulchriceps*, a new species of bruchine beetle of the genus *Acanthoscelides w*hich is under description (Amarillo et al. submitted), and a single individual of *Acanthoscelides macrophthalmus* from Santa Cruz.

**Fig. 2:**
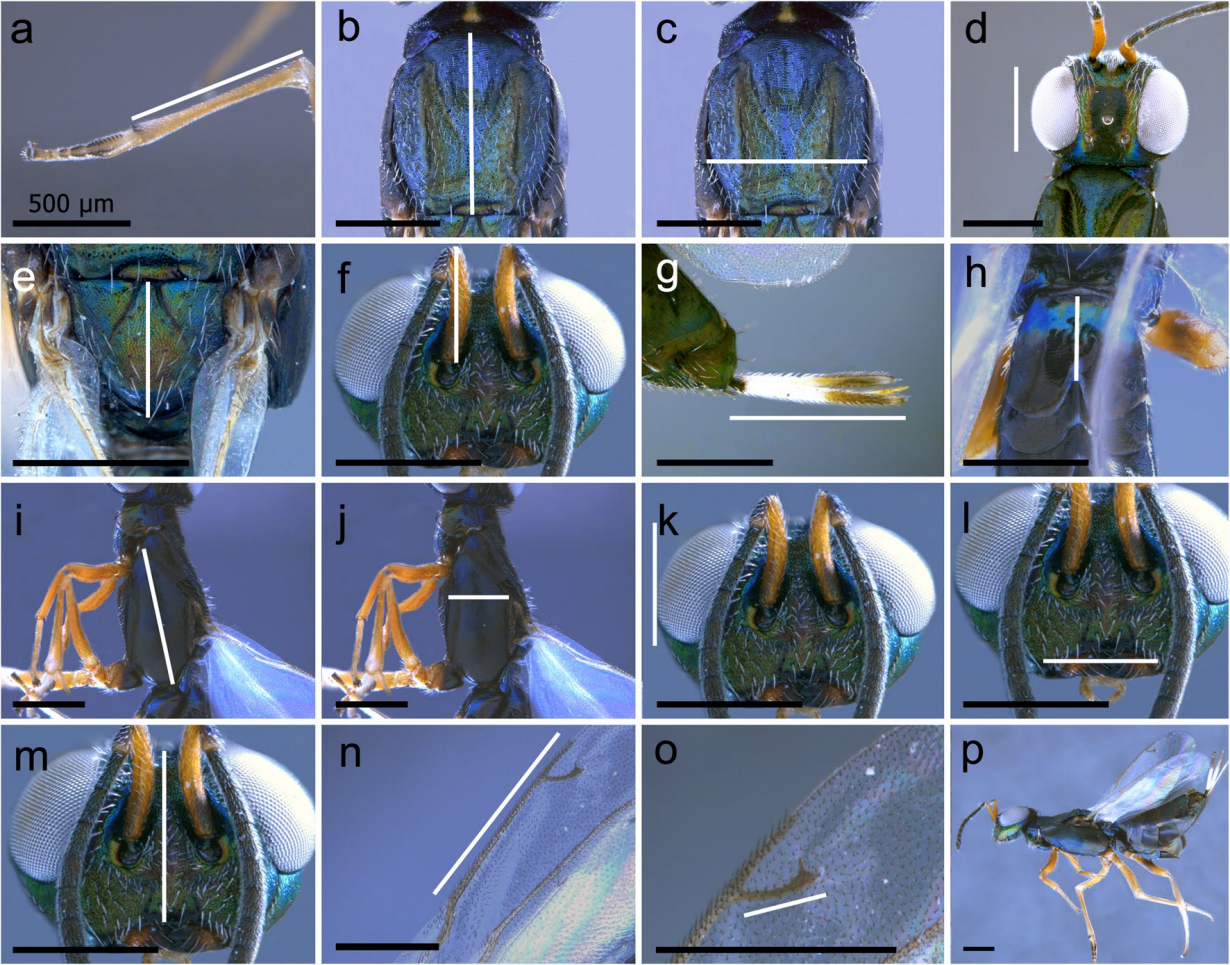
Measurements taken for *Eupelmus pulchriceps* specimens: mesotibia length (**a**), mesoscutum length in dorsal view (**b**), mesoscutum width in dorsal view (**c**), eye length in dorsal view (**d**), scutellum length in dorsal view (**e**), scape length (**f**), ovipositor valve length (**g**), first metasomal tergum length (**h**), mesopleura length (**i**), mesopleura height (**j**), eye length in frontal view (**k**), oral fossa width (**l**), head height in frontal view (**m**), marginal vein length (**n**), stigmal vein length (**o**), *Eupelmus pulchriceps* habitus in lateral view (**p**)

A 64 Megapixel camera attached to a Leica S8AP0 stereoscope was used to photograph the individuals. These were manipulated while submerged in ethanol. Fifteen body structures were measured (Fig. 2), considering their functional relevance (Eggs et al. 2018; Gibson 1986, 1995; Jervis 1998; Segura et al. 2007, Symonds and Elgar 2013). Measurements were taken from the photographs using the software ImageJ (Schneider et al. 2012), and measurement protocol follows Seifert (2002). The structures measured were mesotibia length (Fig. 2a), mesoscutum length in dorsal view (Fig. 2b), mesoscutum width in dorsal view (Fig. 2c), eye length in dorsal view (Fig. 2d), scutellum length in dorsal view (Fig. 2e), scape length (Fig. 2f), ovipositor valve length (Fig. 2g), first metasomal tergum length (Fig. 2h), mesopleura length (Fig. 2i), mesopleura height (Fig. 2j), eye length in frontal view (Fig. 2k), oral fossa width (Fig. 2l), head height in frontal view (Fig. 2m), marginal vein length (Fig. 2n), and the stigmal vein length (Fig. 2o). A general view of a female can be appreciated in Fig. 2p.

Alterations in mesoscutum, mesopleura, and scutellum dimensions reflect modifications related to the mesosoma and, consequently, may provide information on changes about locomotion and the singular jumping strategy of the members of this family (Gibson 1995). The mesotibia is also associated with jumping and locomotive behaviors (Gibson 1986). The marginal and stigmal veins may reflect the size of the wings, which can affect dispersal capacity (Gibson 1986). The size of the eye may be associated with vision and foraging capacity (Segura et al. 2007). The main structures for detecting chemical and auditory signals are the antennae, and their morphological variation is linked to the length of the scape, which could be associated with the type of the host as well as the behavior of the parasitoid (Symonds and Elgar 2013). The oral fossa includes the mouthparts related to the ability to manipulate hosts and feed (Jervis 1998). The ovipositor valve protects the ovipositor and participate in oviposition (Eggs et al. 2018). The length of the first metasomal tergum may be associated with changes in the mobility of the ovipositor.

### Data refinement and variable selection

**Fig. 3:**
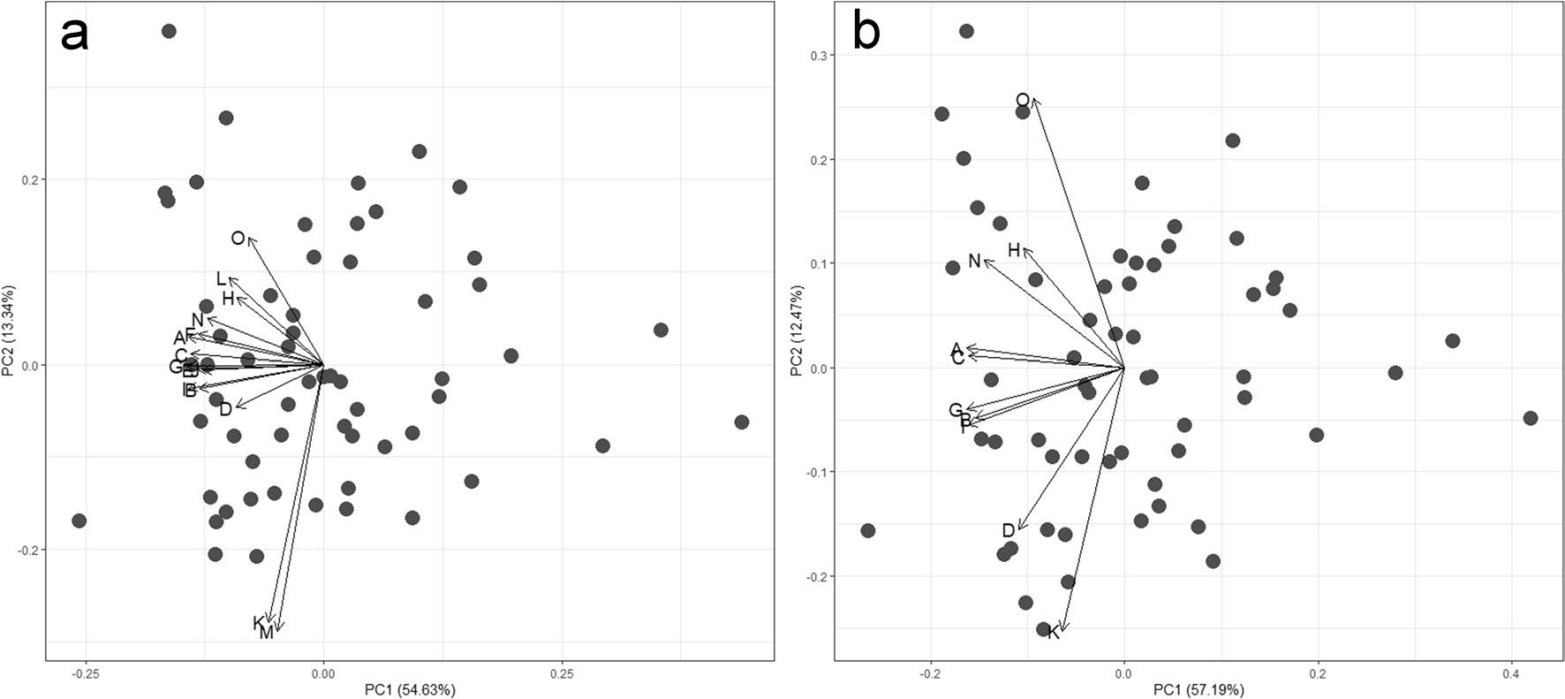
Projection of the first two components of the principal component analysis (PCA) for individuals of *Eupelmus pulchriceps* from the Galapagos islands. The first projection included all the fifteen variables studied (**a**), the second included ten variables after removal five due to redundancy, these are indicated with an asterisk (**b**). Mesotibia length (A), mesoscutum length in dorsal view (B), mesoscutum width in dorsal view (C), eye length in dorsal view (D), scutellum length in dorsal view* (E), scape length* (F), ovipositor valve length (G), first metasomal tergum length (H), mesopleura length (I), mesopleura height* (J), eye length in frontal view (K), oral fossa width* (L), head height in frontal view* (M), marginal vein length (N), and stigmal vein length (O)

A principal component analysis (PCA) was applied to a subsample of 59 individuals and 15 variable measurements per individual with the aim of recognizing the variables that offer redundant information and must be excluded (Fig. 3a and Online Resource) (Mardia et al. 1979). This procedure reduced our dataset to ten variables. To check that this reduction did not alter the morphometric space patterns, we compared the PCA of the complete matrix (15 variables) with that of the reduced matrix (10 variables) (Fig. 3b and Online Resource). The generalized extreme studentized deviate (ESD) test was used to estimate outliers in the reduced matrix (10 variables) (Kuppusamy and Kaliyaperumal 2013) (Online Resource).

### Normality, homoscedasticity, and missing data

Before testing differences within and between islands, normality was evaluated for each variable using either the Shapiro–Wilk test (Royston 1982) or the corrected Kolmogórov-Smirnov test (Dallal and Wilkinson 1986) (Online Resource). Homoscedasticity was also evaluated using Levene’s test (Levene 1960) (Online Resource). Variables that did not meet normality or homoscedasticity were transformed using the Box-Cox method (Box and Cox 1964) (Online Resource). If variables did not meet both parametric requirements despite transformations, we used non-parametric tests. About 1.25% of the database was missing data; these were estimated using Bayesian PCA (Brown et al. 2012). We choose to estimate these data as removing the individuals (15 of 112) implied losing 13% of the sample size. Once these previous phases of data quality assessment were completed, the following tests were conducted with a matrix of 112 females, 40 females from Santa Cruz and 72 from San Cristobal, and ten variables for a total of 1120 measurements.

### Population variation

The variation of individuals was characterized first through a PCA and then through a Sammon mapping technique to determine if the separation is dependent on linear relationships between variables as assumed by standard PCA (Sammon 1969). To evaluate whether the pattern of variation of each island follows the general variation, we compared PCA analyses from each island population and for the entire sample (Online Resource).

To test the morphologic differentiation of populations between islands, we applied MANOVA or PerMANOVA tests depending on compliance with assumptions. We also conducted quadratic discriminant function analysis (QDA) as this method does not require homoscedasticity conformity (Venables and Ripley 2002). To compare the size differences of the wasps between islands, the Mann–Whitney-Wilcoxon two-group non-parametric univariate test was applied to mesotibia length as this variable was the closest associated to the first component of the general PCA (Online Resource) (Mann and Whitney 1947).

To test for differentiation among localities within islands, perMANOVA tests were applied using variables that satisfied the assumption of homoscedasticity. Consequently, we exclude stigmal vein length and mesoscutum length in the dorsal view for San Cristobal; mesopleura length was excluded for Santa Cruz. If differentiation between localities was detected, we tested individual’s membership using QDA.

### Allometric analyses

Differences in the allometric relationships by both slope and height between islands were evaluated. For this, linear regressions were constructed using the mesotibia length as a surrogate of size. All variables were transformed by logarithm base 10. Analyses to test allometric relationships (*slope.test* command) and differences between slopes (*slope.com*) and heights (*elev.com*) were conducted with the specialized package *smatr* of R (Warton et al. 2012). Holm-Bonferroni multiple comparison test was applied to p values.

## Results

### Data refinement and selection of variables

The PCA revealed four groups of variables that offered very similar information; these are group 1: mesotibia length and scape length; group 2: mesoscutum width in dorsal view, scutellum length in dorsal view, ovipositor valve length, and mesopleura height; group 3: first metasomal tergum length and oral fossa width; and group 4: head height in frontal view and eye length in frontal view (Fig. 3a). To avoid redundant information, for future analyses, we selected the variables with the highest loadings on the first or second components within each of the groups mentioned above and excluded other variables that show extreme overlapping (Online Resource). The variables removed are (within brackets is indicated the group these belonged to scape length (1), scutellum length in dorsal view (2), mesopleura height (2), oral fossa width (3), and head height in frontal view (4). In addition, a second PCA was performed to check the effect of removing the redundant variables identified (Fig. 3b). This change reduced the number of variable clusters, and the vectors appeared more dispersed. In this way, the variables cover a good part of the morphological variation and are not affected by collinearity. The ESD test did not identify outliers in any of the variables (Online Resource).

### Population variation between islands

Once we identified the group of variables that described the variation devoid of redundancy, we conducted a PCA to check whether individuals were clustered or distributed evenly across the morphospace. The PCA with the 112 individuals and ten variables followed a similar pattern to that performed with the reduced sample of 59 individuals and 15 variables (Fig. 3b), indicating that neither variable reduction nor sample size affected the general results (Online Resource).

**Fig. 4:**
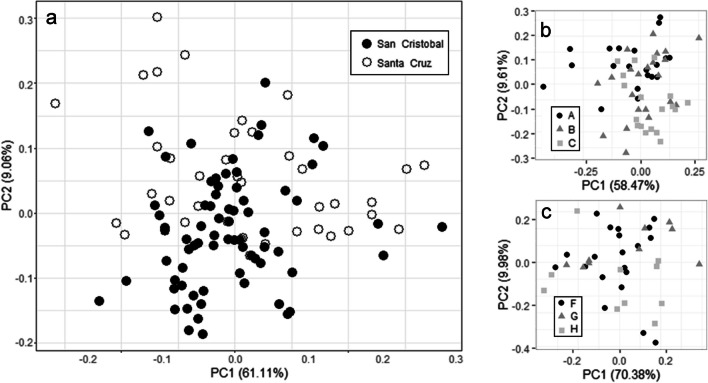
Projection of the individuals of *Eupelmus pulchriceps* from the Galapagos islands in the first two components of the principal components analyses (PCA) discriminating by island (**a**) and by localities; San Cristobal (**b**) and Santa Cruz (**c**). Shape and letters indicate localities within islands

The high proportion of variation explained by the first component (61.1%) indicated that most of the changes between individuals were due to size. The mesotibia length was the variable with the highest eigenvector to the first component (0.374) and, thus, was used as a size descriptor for allometric analyses. However, no difference was observed between island populations when compared only using this structure (Mann–Whitney-Wilcoxon, W = 1555, df = 69.41, sample size = 112, *P* = 0.48). The PCA shows a slight differentiation between islands and no obvious separation by locality within each island (Fig. 4). Due to limits of sampling size in San Cristobal, two localities were excluded. The Sammon Mapping PCA indicated a similar pattern, and thus, traditional PCA analyses were used (Online Resource). The correspondence table of the QDA analysis between islands showed that 73% of the individuals were correctly classified; seven individuals (9.7%) from San Cristobal were classified as from Santa Cruz and seven (17.5%) from Santa Cruz as from San Cristobal. The cross-validation analysis showed no model overfitting as the cases of incorrect identification increased (9.7% vs 18% for San Cristobal and 17.5% vs. 42% for Santa Cruz). Thus, differences between islands were notable (Table 1).

**Table 1:** Classification tables from quadratic discriminant function analysis (QDA) and QDA with cross-validation of the individuals of *Eupelmus pulchriceps* from the Galapagos islands

	Predicted membership	
Original QDA	San Cristobal	Santa Cruz
San Cristobal	65	7
Santa Cruz	7	33
QDA cross-validation		
San Cristobal	59	17
Santa Cruz	13	23
Total	72	40

To test differentiation between islands under more stringent conditions, MANOVA and perMANOVA analyses were performed with the subset of variables that comply assumptions of these tests. These variables were as follows: eye length in dorsal view, eye length in frontal view, and first metasomal tergum length. The MANOVA (Pillai = 0.08, apF = 3.33, df = 1, sample size = 112, *P* = 0.02), and the perMANOVA (*F* = 5.41, *R*^2^ = 0.046, sample size = 112, *P* = 0.005) analyses also showed significant differences between islands. All three structures were larger in San Cristobal than in Santa Cruz but only eye length in dorsal view was statistically different between populations (*T*-test, *F* = 9.49, df = 1, *P* = 0.002).

### Allometric differences between islands

For the entire population of *E. pulchriceps* from the Galapagos, we found significant positive allometry for the mesoscutum length in dorsal view (*b* = 1.33, *P* = 1.00e-05), ovipositor valve length (*b* = 1.24, *P* = 3.27e-05), first metasomal tergum length (*b* = 1.66, *P* = 1.98e-09), and eye length in frontal view (*b* = 1.48, *P* = 1.37e-05). Other structures follow isometric growth (Table 2). When considering the populations from each island, we found common positive allometry for mesoscutum length in dorsal view and first metasomal tergum length (Table 2).

**Table 2:** Allometric slopes calculated for *Eupelmus pulchriceps* populations from each island, and results of slope and height differentiation tests between islands

	San Cristobal	Santa Cruz	Tests for slope differences (*P* value)	Tests for height differences (*P* value)
Mesoscutum length in dorsal view	1.32*	1.36*	0.793	0.388
Mesoscutum width in dorsal view	1.01	0.96	0.689	0.422
Eye length in dorsal view	1.27*	0.83	0.008** (0.05)	0.037** (0.05)
Ovipositor valve length	1.13	1.26*	0.279	0.0002** (0.016)
First metasomal tergum length	1.90*	1.42*	0.085	0.938
Mesopleura length	1.06	1.02	0.707	0.006** (0.025)
Eye length in frontal view	1.71*	1.21	0.071	0.594
Marginal vein length	0.90	1.03	0.200	0.062
Stigmal vein length	1.10	1.01	0.515	4.531e-09** (0.012)

^*^Significant departure of isometry at 0.05; **significant difference between slopes or heights at 0.05. Holm-Bonferroni correction test values are presented within brackets

**Fig. 5:**
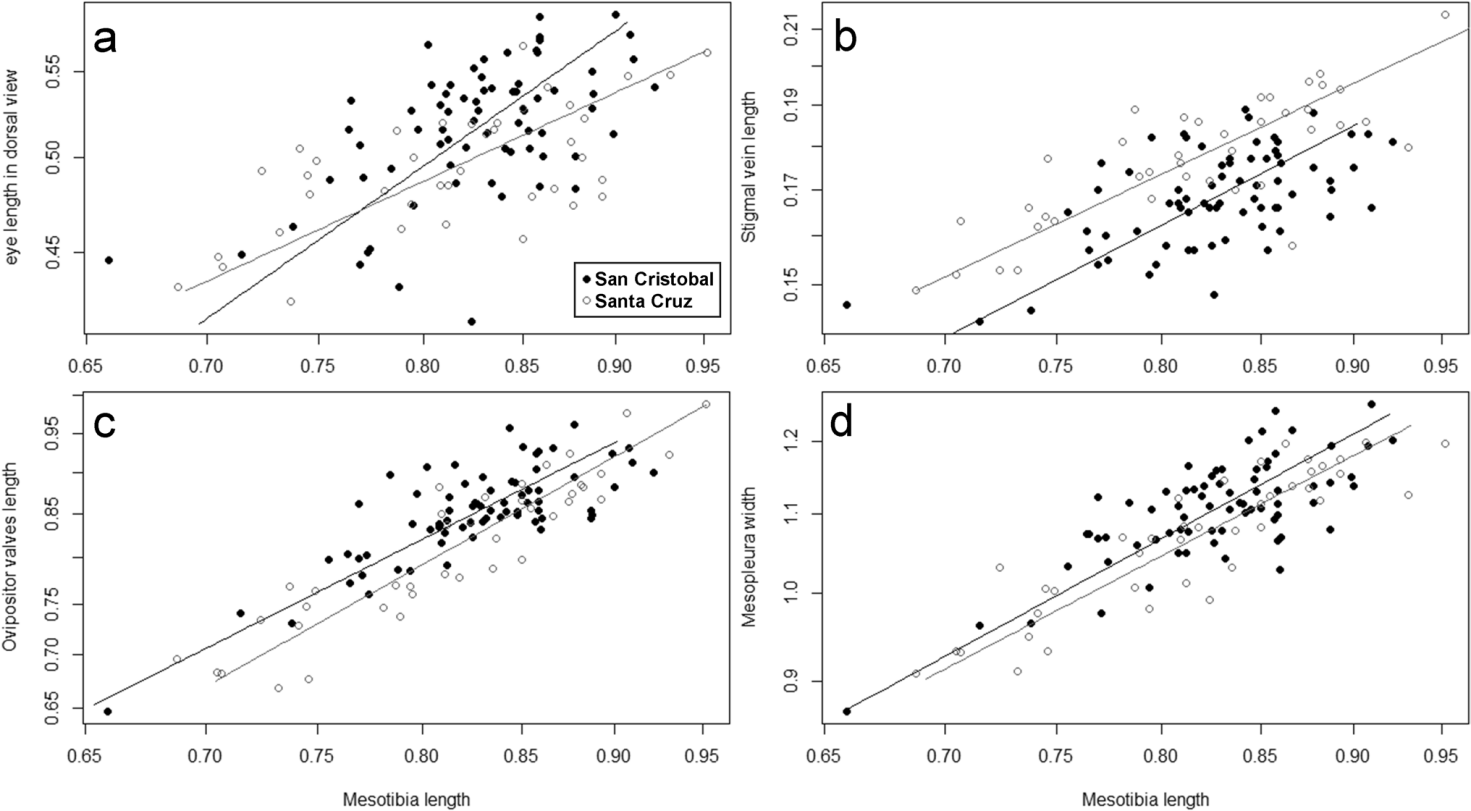
Allometric relationships between body size descriptor and other body variables for *Eupelmus pulchriceps* populations from both islands. Only those exhibiting significant differences between slopes or heights between islands are shown. Mesotibia length was used as body size descriptor (see results, *Population variation*). **a** Eye length in dorsal view, **b** stigmal vein length, **c** ovipositor valve length, and **d** mesopleura length. Black slope and points = San Cristobal Island, Grey = Santa Cruz Island. The log base 10 transformations were applied to both variables

Differences in allometry between islands were detected as follows: positive allometry was identified in the San Cristobal population for eye length in both frontal and dorsal views; in Santa Cruz, it was observed for ovipositor valve length (Table 2). Slope comparisons between populations from the islands showed differences only for eye length in dorsal view (*P* = 0.008, Table 2), with a steeper slope for the San Cristobal population (Fig. 5a). Four variables differed between islands for slope elevation or height, eye length in dorsal view, ovipositor valve length, mesopleura length, and stigmal vein length (Table 2). However, the trends were not uniform: eye length in dorsal view, mesopleura length, and ovipositor valve length relationships were higher for San Cristobal, and stigmal vein length was higher for Santa Cruz (Fig. 5).

### Comparison of populations from San Cristobal Island

The first principal component explained 58.47% and the second 9.61% of the variation (Online Resource), indicating that a substantial portion of the variation was due to changes in size. Individuals from locations A and C were slightly clustered (Fig. 4b). The projection with Sammon mapping showed a similar trend (Online Resource), suggesting that regular PCA adequately expressed morphometric variation. In the QDA, we found that 40.9% of individuals were correctly identified; two individuals (10%) from A were classified in B, one from A in C (5%) (Table 3), and one from B in C (4.3%). The cross-validation analysis indicated no over-adjustment of the model (10% vs. 55% for A in B, 5% vs. 20% for A in C, and 4.3% vs. 4.3% B in C). The perMANOVA analyses indicated significant differences between populations (*F* = 2.70, *R*^2^ = 0.85, sample size = 70, *P* = 0.043), with the post hoc test showing significant differences between sites A and C (*F* = 4.81, *R*^2^ = 0.11, sample size = 70, *P* = 0.045). Thus, comparing these results with those between islands showed that the differences among San Cristobal populations were not as apparent.

**Table 3:** Classification tables from quadratic discriminant function analysis (QDA) and QDA with cross-validation of the individuals of *Eupelmus pulchriceps* from San Cristobal Island. Localities are indicated by letters; see map of Fig. 1 for location

	Predicted membership		
Original QDA	A	B	C
A	17	2	1
B	2	20	1
C	1	1	16
QDA cross-validation			
A	5	9	6
B	11	13	5
C	4	1	7
Total	20	23	18

### Comparison of populations from Santa Cruz Island

The first component of the PCA explained 70.38%, and the second explained 9.98% of the variation, indicating that a large portion of the variation was due to changes in size. The variables from the Santa Cruz population behaved differently from those from San Cristobal and even from the total sample of the archipelago (Online Resource). There was no separation between individuals from the three locations of the island (Fig. 4c). The projection with Sammon mapping showed a similar trend (Online Resource). The perMANOVA (*F* = 0.57, *R*^2^ = 0.03, sample size = 42, *P* = 0.64) analysis indicated no differences between populations.

## Discussion

### Differentiation in Eupelmus pulchriceps populations

We found multivariate statistical differences in linear measurements between the populations of *E. pulchriceps* from the islands of Santa Cruz and San Cristobal; however, no difference was observed when using mesotibial length as single indicator (Mann–Whitney-Wilcoxon was applied, *P* = 0.48) which is the variable closely related to size. These results indicate that populations are different by a combination of traits rather than by changes in body size. In comparison, the populations between locations within islands shows small or no differences; the only case of within island differentiation was detected between the more distant locations of San Cristobal (1400 m) but no between the more distanced sites in Santa Cruz (2000 m) despite being further apart in the latter. We can attribute this general result to the mobility of the wasps within the islands being the single case a false positive; future sampling may solve this result. Our overall finding is consistent with the greater distance between the islands (80.93 km) than between the localities. In San Cristobal, the average distance between localities was 1.13 km (max = 1.44 km), and in Santa Cruz, it was 1.3 km (max = 1.96 km). These results follow studies where morphological differentiation has been associated with the distance or age of the islands, even to the point where lineages are defined as different species (Hollocher 1996; Brunton and Hurst 1998; Gillespie and Roderick 2002), including short periods of time (Chamberland et al. 2020), or as low as 20 generations such as described by Pascoal et al. (2019) studying adaptation and disruptive selection in the cricket *Teleogryllus oceanicus* (Le Guillou, 1841). Thus, despite differences in size and shape may be imperceptible to the naked eye, these can reflect ongoing significant evolutionary events (Pfingstl and Baumann 2017) or local adaptation (MacLean et al. 2016; Frazier et al. 2008; Rohner and Moczek 2020).

In general, the changes observed in *E. pulchriceps* populations agree with findings in related studies. In addition to an overall positive allometry towards body elongation (mesoscutum and first metasomal tergum length) in the entire sample of the islands, and agreeing with many studies (Houle et al. 2019), we found more differences for heights (4) than for slopes (1) between the populations of *E. pulchriceps* from the two islands. These differing trends between populations suggest ongoing differentiation (Voje et al. 2014; Rohner 2020), and although static allometry has been considered a constraint for the evolution of traits, Voje et al. (2014) report a close link between static allometry and evolutionary allometry for multiple species; similarly, Houle et al. (2019) in their review, and Frankino et al. (2007) in their study of the butterfly *Bicyclus anynana* (Butler, 1879) reported that allometric changes can be affected by natural selection even in the short term. Mesoscutum width in dorsal view and marginal vein length always followed an isometric pattern and never differed between and within islands in either slope or height, suggesting high conservatism in these characters. This result agrees with a long-stated tradition of using mesoscutum width as a body size proxy for hymenopterans (Ohl and Thiele 2007). It can be intriguing to the reader that we used mesotibial length as body size descriptor instead but as we described in the results both variables were extremely close in the PCA and the loadings of the latter were slightly superior (Online resources). Marginal vein length may be linked to mesoscutum width as these structures are associated with flight. The projection of these variables in the general PCA shows closeness between variables (Online Resource). Eye length in the dorsal view showed differences in slope and heights between islands, suggesting that the structure is prone to change. This result is consistent with Al khatib et al. (2014), who repeatedly use eye height for differentiation between species of the *Eupelmus urozonus* complex. It also indirectly agrees with Askew and Nieves-Aldrey (2017), who use head height in their taxonomic keys of *Calosoma* (Eupelmidae). Height differences between the populations in three measurements (stigmal vein length, ovipositor valve length, and mesopleura length) follow a different pattern; the first is higher in Santa Cruz, while the other two structures show higher values in San Cristobal. These results support shape differentiation, as has been shown in studies with different animal groups (Beheregaray et al. 2004; Krolow et al. 2020; Mahler et al. 2010; Pfingstl and Baumann 2017; VanderWerf et al. 2009). Different allometric responses by distinct populations of the same species have been reported in those cases (Amshokova 2010; Baláž et al. 2012; Sota et al. 2000). The ovipositor valve length is identified as an evolutionarily labile trait in other *Eupelmus* species (Al Khatib et al. 2016), potentially associated with changes in hosts (Eggs et al. 2018). We reiterate that these changes in relative growth can indicate selective pressures, either as a general trend or as a differential factor between populations (Frankino et al. 2005; Kawano 2002; Lane 1981).

We did not measure the genetic structure of the populations, and thus, we cannot rule out that we are recording phenotypic plasticity responses which may be seen as accidental events in the evolution of the group. We also acknowledge the importance of detecting this line of evidence that may have significant consequences in our conclusions (Prada et al. 2008). However, we believe that consistent differentiation processes, even without genetic changes, may place each population in a different part of their morphospace and thus may facilitate evolutionary events. A growing number of publications have pointed out to the acknowledgement of phenotypic plasticity as an initial force to trigger lineage disruptions (West-Eberhard 2005; Pfennig et al. 2010; Casasa and Moczek 2019). As Fusco and Minelli (2010) point out, environmental and genetic components can no longer be seen as separate or opposite factors in evolution but as boosters of variation that potentially affects evolution.

Given that samples from each island came from different years, it can be argued that environmental conditions may explain differences between islands as these can influence body dimensions (Zhang et al. 2019, 2021); however, only rainfall showed an important reduction (≈ 30%) between 2018 and 2019. If we take this into consideration, we may expect significant changes in body size. Instead, as we described, differentiations were detected only considering multivariate analyses and body size values were statistically indistinguishable.

### Eupelmus pulchriceps as a foreign species

Studying differentiation in the early stages of a contemporary time scale is essential for understanding the dynamics of a foreign species in a new area as it offers an opportunity to unravel ecological and environmental interactions and its evolutionary consequences (Colautti and Lau 2015). Contemporary differentiation of populations can occur both as an adaptative response to natural selection and through stochastic changes resulting from the history of the introduction, founder effects, or genetic drift (Colautti and Lau 2015). To insert our case within the context of these processes, we need to document three aspects: time of arrival, history of dispersion in the islands, and the number of generations occurring over these populations.

We estimated that the presence of *E. pulchriceps* in the Galapagos is due to a single recent event based on the following lines of evidence: (1) The host plant of the bruchine beetle host was intentionally introduced to Santa Cruz Island in 1985 and later to San Cristobal (Rentería et al. 2007); posteriorly, it was reported in the islands of Isabela and Floreana but intensive eradication efforts have left a few plants in these two islands; no record of the plant has been registered in other islands (Bungartz et al. 2023; Amarillo-Suarez et al. 2020). (2) Despite a sampling effort of over 550 kg of seeds where several exotic legume plants present in four islands of the archipelago were included (Amarillo-Suarez et al. 2020), *E. pulchriceps* was found only in *L. leucocephala* on the two mentioned islands. (3) A recent thorough structured sampling of Hymenoptera conducted along 3 years using several sampling methods in the thirteen major islands of the archipelago that significantly increased the number of species recorded from 84 to over 399 species (Picón-Rentería et al. in prep.) did not provide occurrences of *E. pulchriceps* out of Santa Cruz and San Cristobal. Finally, 4 Santa Cruz and San Cristobal are the more populated islands with the highest levels of human transformations and connectivity due human routine travel (Reyes et al. 2016; Reyes et al. 2017; Páez-Rosas and Guevara 2017; Lasso and Espinosa 2017; Delgado 2018).

These arguments appear convincing enough to our statement, but we acknowledge that there are other options, for example, the presence of the wasp on other islands could challenge our proposed relationship between morphological differentiation and distances, unfortunately, as we described above, there are no records as far as we know. Likewise, an independent colonization of the islands due earlier and already differentiated populations may provide a false reading of our results; this hypothetical scenario requires further research including information of continental populations and phylogeographical analysis with molecular data although the history of arrival of the plant suggest a single colonization event.

A final argument to suggest the recent nature of the arrival deals with the type of differentiation of the populations of *E. pulchriceps*. This is present for some structures as an admixture of changes within islands, not as a consistent difference in all structures. Differentiation is not uniform for all structures. In several cases, it is associated with the relevant functional structures, possibly responding to each island’s different ecological and environmental pressures (Al khatib et al. 2016). We also noticed that labile structures of *E. pulchriceps* on the islands had shown similar behaviors in other geographical regions, suggesting a certain predictability of the changes expected for founding populations of this species.

Based on the following information we estimate that *E. pulchriceps* has been in the archipelago enough time to experience differentiation. The life cycle of *E. pulchriceps* takes about 23 days at 26 °C ± 2 °C and 30 ± 5% RH (González 2017), which is among the lower range for the species of the genus (*E. messene* 25 days, *E. microzonus* 27 days, (Gokhman and Nikelshparg 2021) and *E. orientalis* 20 days (Doury and Rojas-Rousse (1994)). Reproduction for many insects in the Galapagos is limited to the rainy season which may occur at most during six months per year (Peck 2008); given the time of arrival of *L. leucocephala* to the islands, and with the optimistic assumption of an almost simultaneous colonization of the beetle host and the wasp to the archipelago, about 272 generations of the wasp may have been produced. Several studies have demonstrated differentiation or adaptation processes in insects with even lower number of generations: Gomi (2007) detected adaptation in the moth *Hyphantria cunea* after 120 generations; Pascoal et al. (2019) identified adaptation in the cricket *Teleogryllus oceanicus* after just 20 generations. Finally, Tomasetto et al. (2017) documented evolution of resistant populations to pesticides after 50 generations in the beetle *Listronotus bonariensis* (Kuschel, 1955).

We would like to call attention to an interesting observation: *Eupelmus pulchriceps* is only found parasitizing a single and new species of bruchine beetle that consumes the seeds of the invasive species *L. leucocephala* in both islands (Amarillo et al. submitted). This is important because we report population differentiation and thus potential local adaptation of an exotic species. Apparently, the wasps’ populations currently depend on a single beetle species for their survival; however, due to the wasps’ polyphagy (Gibson 1995; Stireman 2005) and the presence of several other species of seed predator beetles in the plant (Amarillo et al. submitted), its invasive potential is high (Kolar and Lodge 2001); thus, it is likely that *E. pulchriceps* can parasitize new hosts and sustain its population in other trophic webs modifying local species interactions and nutrient dynamics (Colautti and Lau 2015; Stigall 2016; Chaves 2018; Keith et al. 2016). Such has been the case in Hawaii, where *E. pulchriceps* spread rapidly to local beetles after a recent introduction (Stein 1983).

### Supplementary Information

Below is the link to the electronic supplementary material.

Supplementary file1 (DOCX 6159 KB)

## Data Availability

Data available on request from the authors.
