# Supplementary material for: Morphologic Differentiation of the Exotic Parasitoid Eupelmus pulchriceps (Hymenoptera: Eupelmidae) in the Galapagos Archipelago
Source: Neotrop Entomol. 2023 Dec 22;53(1):140–53. doi: 10.1007/s13744-023-01097-3 (PMC10834596; doi:10.1007/s13744-023-01097-3)
Supplement: Supplementary file 1 — Supplementary file1 (DOCX 6159 KB) [file 13744_2023_1097_MOESM1_ESM.docx]

**Title**

**Morphologic differentiation of the exotic parasitoid Eupelmus pulchriceps (Hymenoptera: Eupelmidae) in the Galapagos archipelago.**

**Neotropical Entomology**

**Author information**

Nicolas David Camargo-Martinez*, Laboratorio de Sistemática y Biología Comparada de Insectos. Instituto de Ciencias Naturales, Universidad Nacional de Colombia. Bogotá, Colombia.
***Corresponding autor:** [ndcamargom@unal.edu.co](mailto:ndcamargom@unal.edu.co)
https://orcid.org/0009-0009-1935-3213

Mariana Camacho-Erazo, Museo de Entomología, Facultad de Recursos Naturales. Escuela Superior Politécnica del Chimborazo. Riobamba, Ecuador. http://orcid.org/0000-0001-8591-0254

Angela R. Amarillo-Suárez, Departamento de Ecología y Territorio, Facultad de Estudios Ambientales y Rurales, Pontificia Universidad Javeriana. Bogotá, Colombia. https://orcid.org/0000-0001-9904-0202

Henri W. Herrera, Museo de Entomología, Facultad de Recursos Naturales. Escuela Superior Politécnica del Chimborazo. Riobamba, Ecuador. https://orcid.org/0000-0002-1417-4349

Carlos E. Sarmiento, Laboratorio de Sistemática y Biología Comparada de Insectos. Instituto de Ciencias Naturales, Universidad Nacional de Colombia. Bogotá, Colombia. https://orcid.org/0000-0003-4012-8108

**Online Resource.**


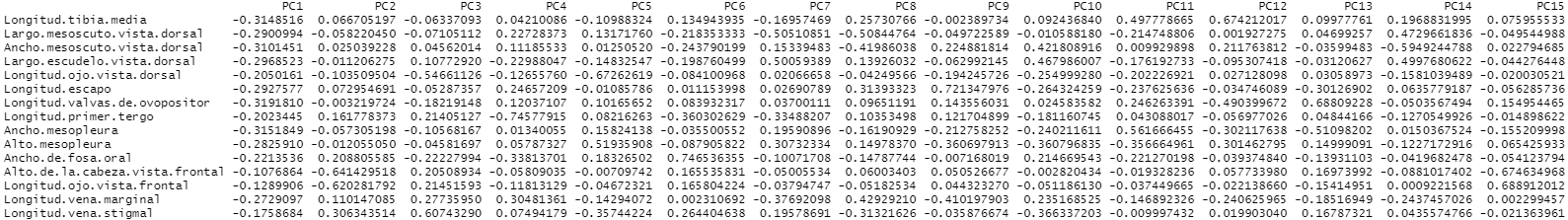

**Table S1.** PCA loads with 59 individuals and 15 variables.


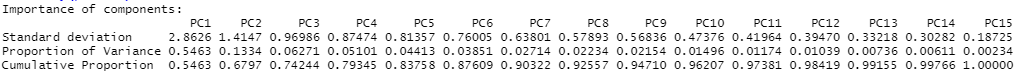


**Table S2.** Proportion of the accumulated variance of PCA with 59 individuals and 15 variables.


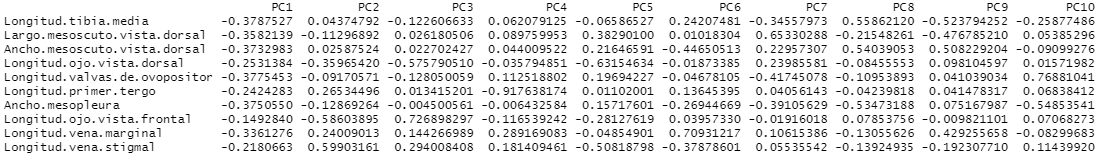

**Table S3.** PCA loads with 59 individuals and ten variables.

**
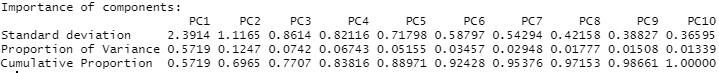
**
**Table S4.** Proportion of the cumulative variance of PCA with 59 individuals and ten variables.

Mesotibia length

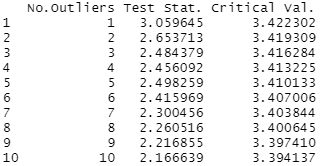


Mesoscutum length in dorsal view

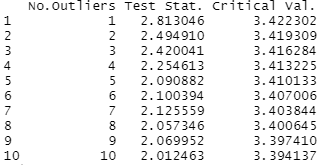


Mesoscutum width in dorsal view.


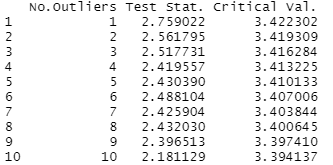


Eye length in dorsal view.


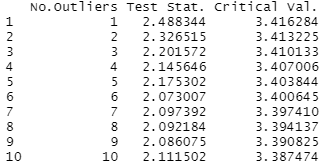


Length of ovipositor valves.


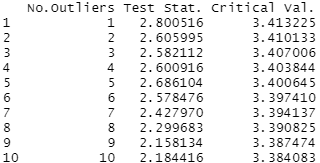


Length of first metasomal tergum.


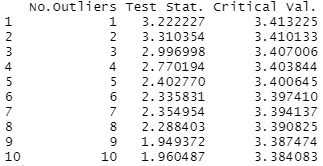


Length of mesopleura.


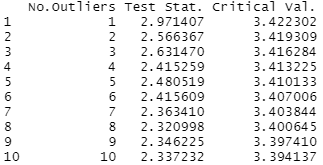


Length of the eye in frontal view.


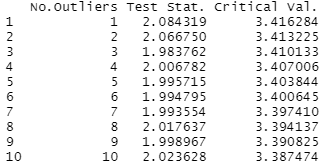


Length of the marginal vein.


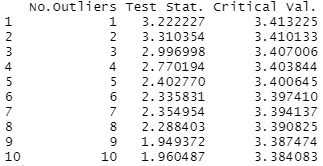


Length of the stigmal vein.


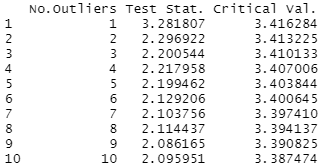


**Table S5.** Values of the generalized extreme studentized deviate (ESD) test for mesotibia length, mesoscutum length in dorsal view, mesoscutum width in dorsal view, eye length in dorsal view, length of ovipositor valves, length of first metasomal tergum, length of mesopleura, length of the eye in frontal view, length of the marginal vein and length of the stigmal vein. When the value of the test statistic is greater than the critical value, we recognize that the number of outliers for the variable is found at that point.


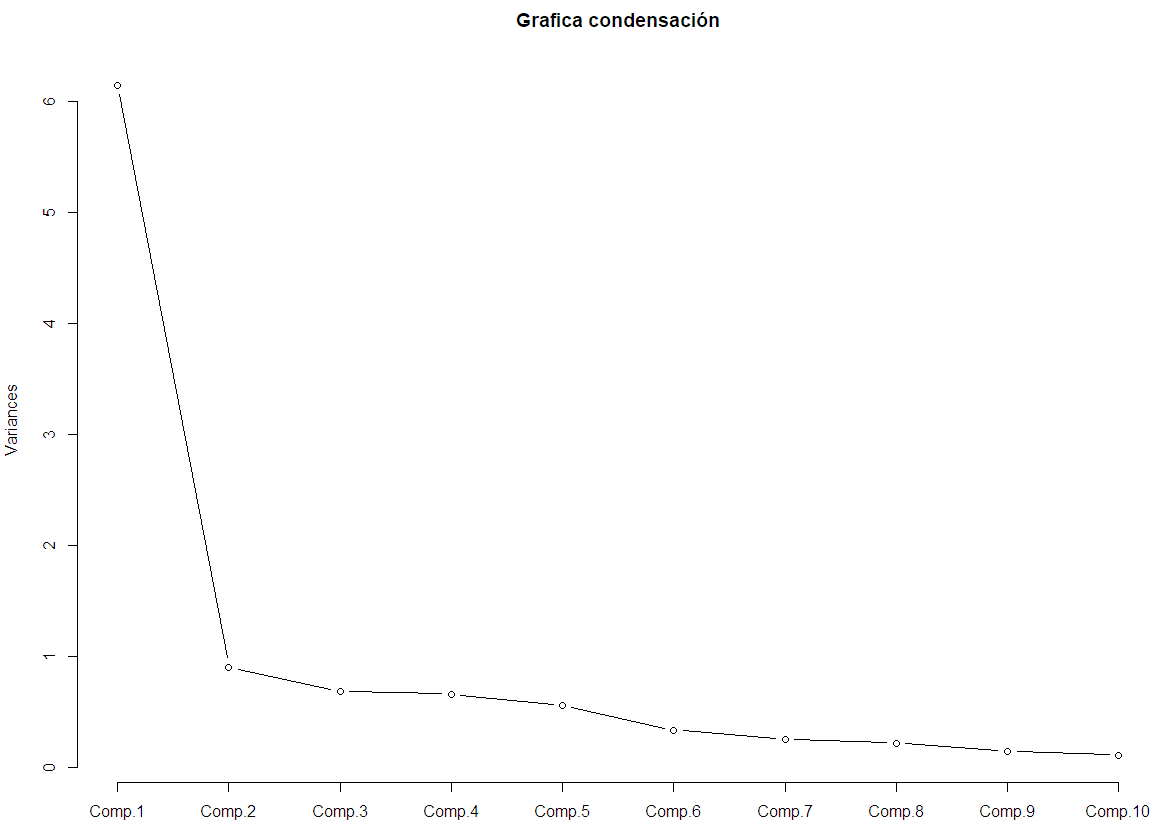

**Figure S1.** Condensation graph and PCA with 112 individuals and ten variables.

*
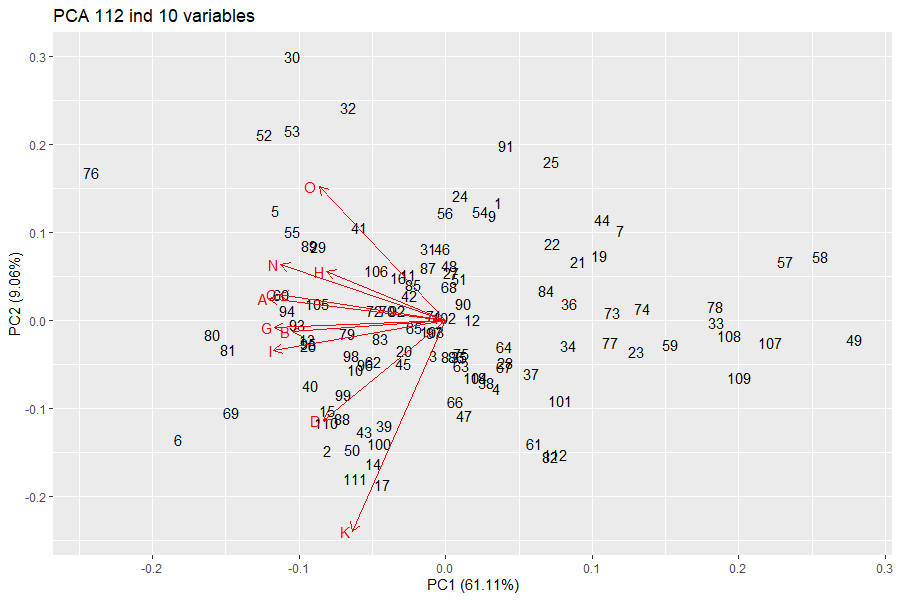
***Figure S2.** Projection of the first two PCA components with 112 individuals and ten variables: mesotibia length (A), mesoscutum length in dorsal view (B), mesoscutum width in dorsal view (C), eye length in dorsal view (D), length of ovipositor valves (G), length of first metasomal tergum (H), length of mesopleura (I), length of the eye in frontal view (K), length of the marginal vein (N) and length of the stigmal vein (O).


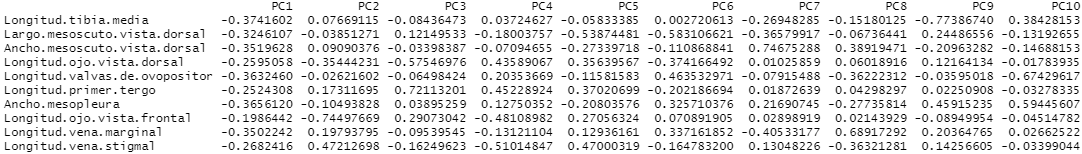
 **Table S6.** PCA loads with 112 individuals and 10 variables.

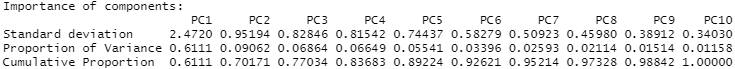
  **Table S7.** Proportion of variance and cumulative proportion of PCA with 112 individuals and ten variables.

**
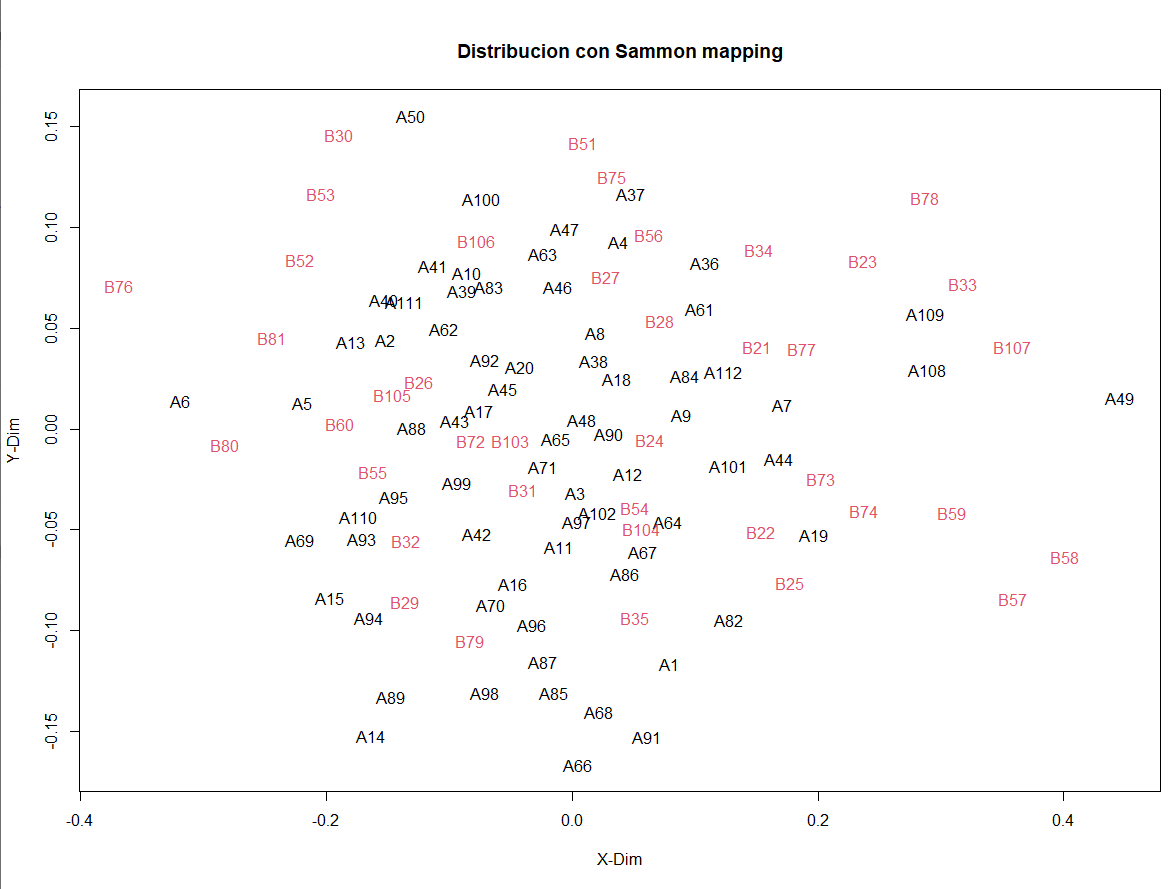
**

**Figure S3.** Projection of the individuals in the first two components of a PCA type Sammon mapping with 112 individuals and ten variables discriminated by islands A= San Cristobal, B= Santa Cruz.


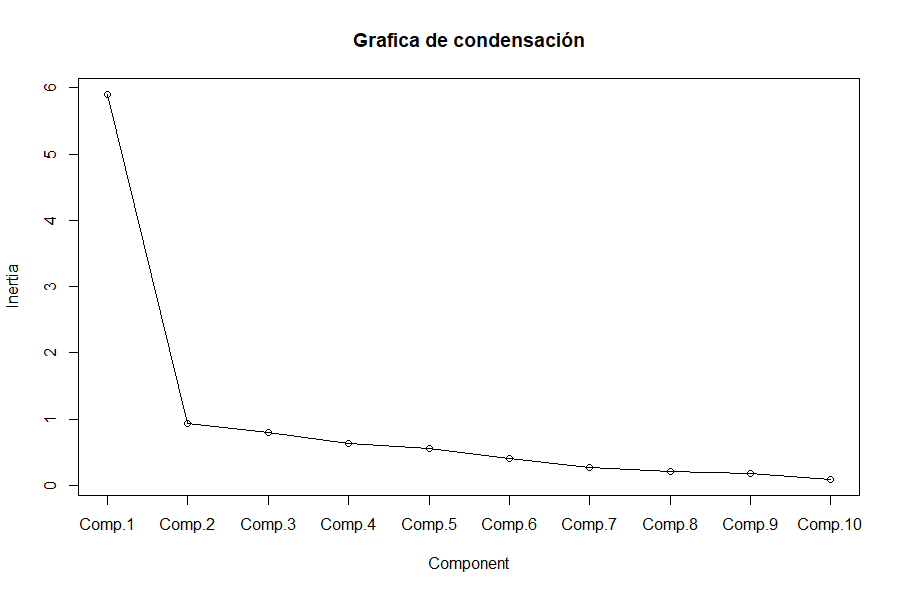


**Figure S4.** Condensation graph of San Cristobal Island with 61 individuals and ten variables.


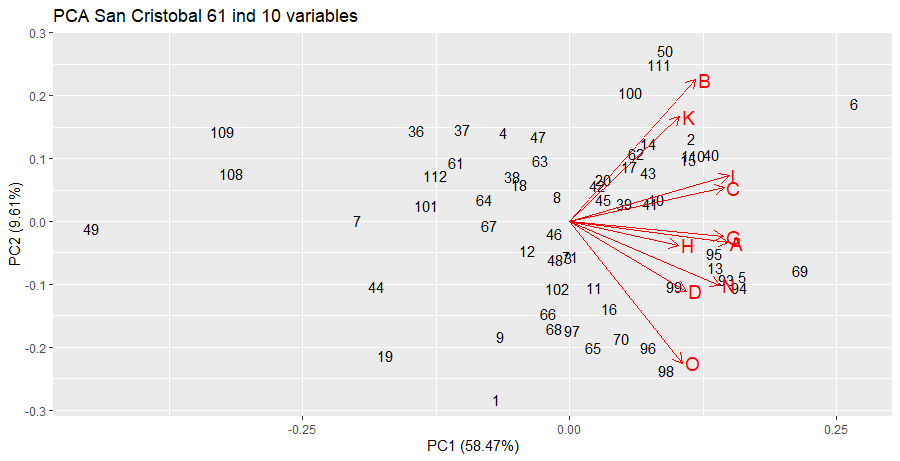


**Figure S5.** Principal component analysis (PCA) from San Cristobal Island population: mesotibia length (A), mesoscutum length in dorsal view (B), mesoscutum width in dorsal view (C), eye length in dorsal view (D), length of ovipositor valves (G), length of first metasomal tergum (H), length of mesopleura (I), length of the eye in frontal view (K), length of the marginal vein, (N) and length of the stigmal vein (O).


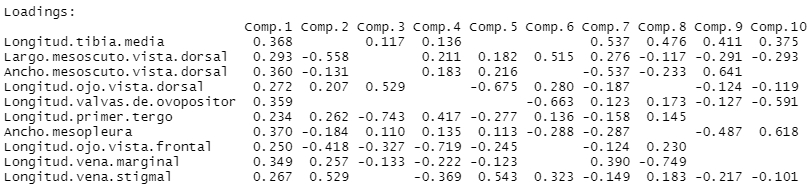


**Table S8.** PCA loads with 61 individuals and ten variables from San Cristobal.


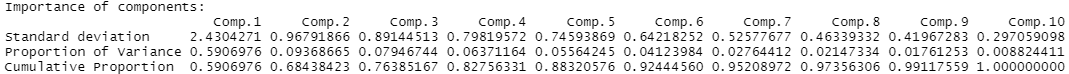


**Table S9.** Proportion of the cumulative variance of PCA with 61 individuals and ten variables from San Cristobal.
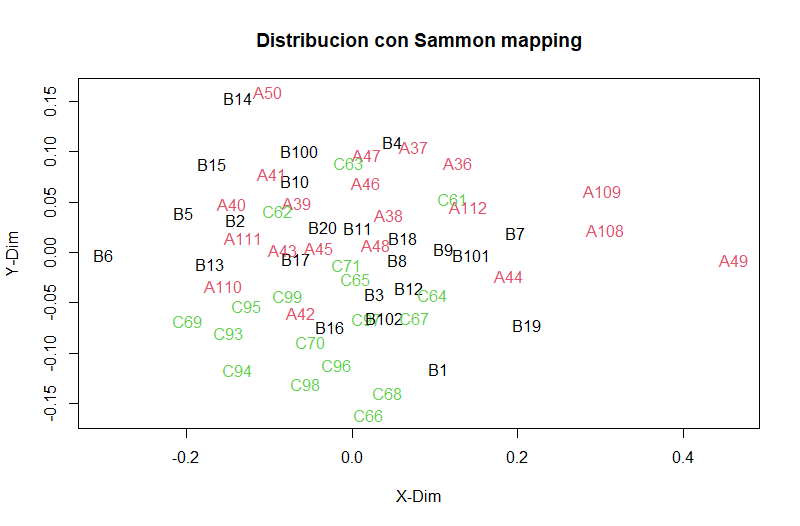


**Figure S6.** Projection of the individuals in the first two components of a PCA type Sammon mapping discriminated by locations on San Cristobal.

**
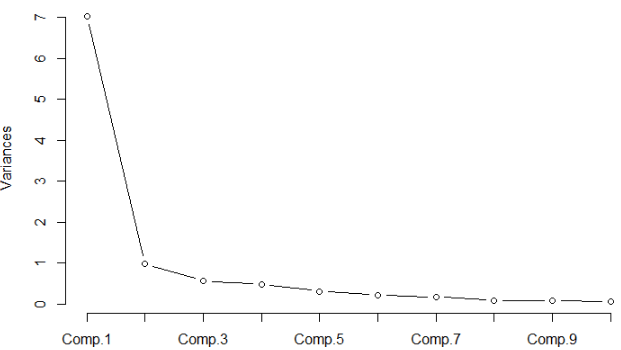
**

**Figure S7.** Condensation graph of Santa Cruz Island with 40 individuals and ten variables.

**
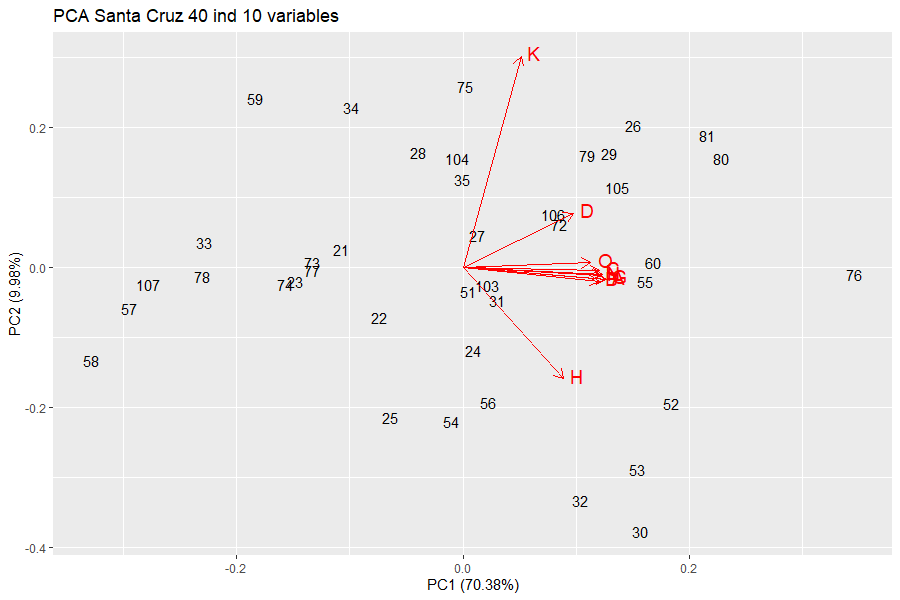
**

**Figure S8.** Principal component analysis (PCA) from Santa Cruz Island: mesotibia length (A), mesoscutum length in dorsal view (B), mesoscutum width in dorsal view (C), eye length in dorsal view (D), length of ovipositor valves (G), length of first metasomal tergum (H), length of mesopleura (I), length of the eye in frontal view (K), length of the marginal vein, (N) and length of the stigmal vein (O).


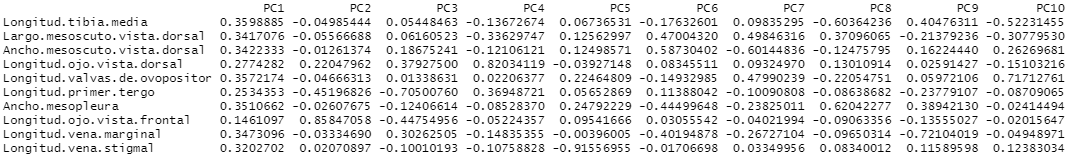


**Table S10.** PCA loads with 40 individuals and ten variables.


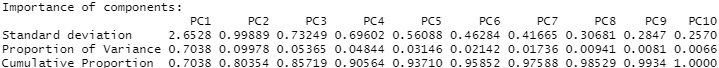


**Table S11.** Proportion of the cumulative variance of PCA with 40 individuals and ten variables.

**
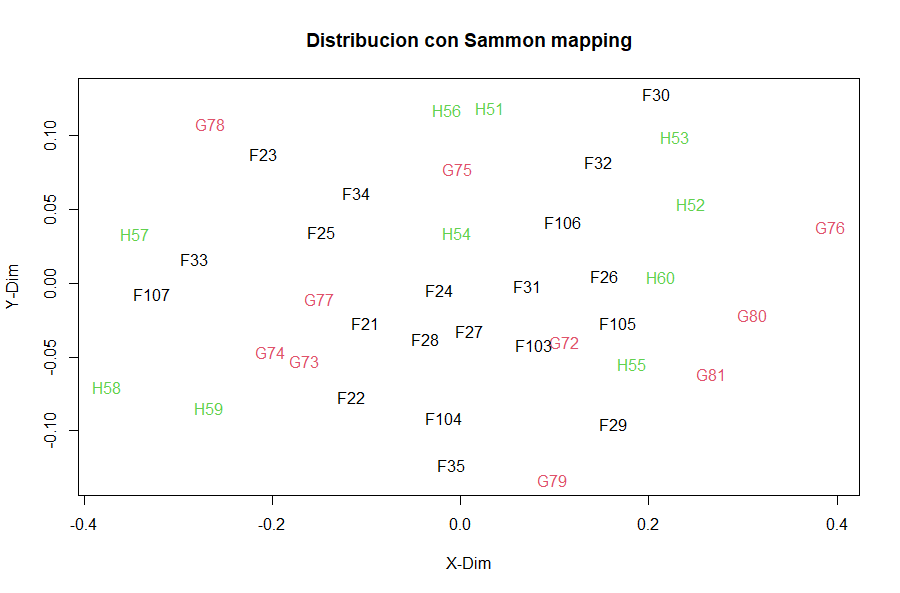
**

**Figure S9.** Projection of the individuals in the first two components of a PCA type Sammon mapping discriminated by locations of Santa Cruz.

*Archipelago of Galapagos.*

*Evaluation of normality and homoscedasticity*

In general, the variables that do not present a normal distribution (P<0.05) are the same for the Shapiro-Wilk and Kolmogórov-Smirnov tests (Table 1). These are the: length of the ovipositor valves, length of the first tergum and length of the mesopleura. However, there is a discrepancy in one variable; Shapiro indicates normality for the length of the mesotibia while Kolmogórov-Smirnov does not.

On the other hand, the variables that meet the homoscedasticity requirement (P<0.05) are the length of the eye in dorsal view, length of the first tergum, and length of the eye in frontal view.

|  | Shapiro-Wilk | Kolmogórov-Smirnov | Levene | Box-Cox | Shapiro-Wilk | Kolmogórov-Smirnov | Levene |
| --- | --- | --- | --- | --- | --- | --- | --- |
| Mesotibia length | 0.161 | 0.032 | 0.003 | Yes | 0.983 | 0.459 | 0.004 |
| Mesoscutum length in dorsal view | 0.957 | 0.708 | 0.002 | Yes | 0.978 | 0.889 | 0.002 |
| Mesoscutum width in dorsal view | 0.302 | 0.846 | 0.028 | Yes | 0.913 | 0.941 | 0.029 |
| Eye length in dorsal view | 0.312 | 0.4101 | 0.638 | No |  |  |  |
| Length of ovipositor valves | 0.005 | 0.00003 | 4.4e-05 | Yes | 0.348 | 0.017 | 0.0005 |
| Length of first metasomal tergum | 0.013 | 0.042 | 0.529 | Yes | 0.051 | 0.373 | 0.707 |
| Length of mesopleura | 0.013 | 0.018 | 0.005 | Yes | 0.812 | 0.605 | 0.037 |
| Length of the eye in frontal view | 0.230 | 0.957 | 0.987 | No |  |  |  |
| Length of the marginal vein | 0.054 | 0.075 | 0.001 | Yes | 0.279 | 0.221 | 0.001 |
| Length of the stigmal vein | 0.796 | 0.801 | 0.022 | Yes | 0.926 | 0.922 | 0.046 |

**Table S12.** P-values of Shapiro-Wilk and Kolmogórov-Smirnov normality tests and P-values of Levene's homoscedasticity test. Box-Cox transformed variables are also presented.

Thus, the Box-Cox transformation was performed for all the variables that did not show normality or homoscedasticity, including the variable that showed discrepancies by tests. According to Shapiro-Wilk, all the variables adjusted to a normal distribution. On the other hand, for Kolmogórov-Smirnov only the length of the ovipositor valves did not fit a normal distribution. Due to the discrepancies between tests, we rely on Shapiro-Wilk since it is the most powerful for evaluating the normal distribution of the variables regardless of their particularities (Yap & Sim, 2011). Finally, according to Levene (1960), no variable was adjusted to homoscedasticity.

*San Cristobal*

*Evaluation of normality and homoscedasticity*

The variables that do not present a normal distribution (P <0.05) are: the length of the mesotibia, length of the ovipositor valves, and length of the mesopleura. The variables that do not present homoscedasticity (P<0.05) are the: length of the mesotibia and the length of the first tergus. Thus, the Box-Cox transformation was performed for these variables. According to Shapiro-Wilk and Kolmogórov-Smirnov, all adjusted to a normal distribution. Finally, according to Levene (1960), no variable was adjusted to homoscedasticity.

|  | Shapiro-Wilk | Kolmogórov-Smirnov | Levene | Box-Cox | Shapiro-Wilk | Kolmogórov-Smirnov | Levene |
| --- | --- | --- | --- | --- | --- | --- | --- |
| Mesotibia length | 0.0682 | 0.364 | 0.347 | No |  |  |  |
| Mesoscutum length in dorsal view | 0.7397 | 0.6419 | 0.525 | No |  |  |  |
| Mesoscutum width in dorsal view | 0.2325 | 0.7332 | 0.038 | Yes | 0.9774 | 0.9572 | 0.0389 |
| Eye length in dorsal view | 0.1692 | 0.2559 | 0.518 | No |  |  |  |
| Length of ovipositor valves | 0.011 | 0.01591 | 0.0685 | Yes | 0.598 | 0.152 | 0.338 |
| Length of first metasomal tergum | 0.1593 | 0.764 | 0.302 | No |  |  |  |
| Length of mesopleura | 0.1021 | 0.4761 | 0.11 | No |  |  |  |
| Length of the eye in frontal view | 0.4227 | 0.7222 | 0.286 | No |  |  |  |
| Length of the marginal vein | 0.1471 | 0.3012 | 0.193 | No |  |  |  |
| Length of the stigmal vein | 0.3171 | 0.5269 | 0.045 | Yes | 0.4577 | 0.5856 | 0.0352 |

**Table S13.** P-values of Shapiro-Wilk and Kolmogórov-Smirnov normality tests and P-values of Levene's homoscedasticity test according to San Cristobal Island populations. The variables transformed by Box-Cox are also presented.

*Santa Cruz Island.*

*Evaluation of normality and homoscedasticity*

The variable that does not present normal distribution and homoscedasticity (P<0.05) is the length of the first tergum. And only this variable underwent Box-Cox transformation.

|  | Shapiro-Wilk | Kolmogórov-Smirnov | Levene | Box-Cox | Shapiro-Wilk | Kolmogórov-Smirnov | Levene |
| --- | --- | --- | --- | --- | --- | --- | --- |
| Mesotibia length | 0.552 | 0.367 | 0.213 | No |  |  |  |
| Mesoscutum length in dorsal view | 0.843 | 0.813 | 0.0571 | No |  |  |  |
| Mesoscutum width in dorsal view | 0.803 | 0.868 | 0.0756 | No |  |  |  |
| Eye length in dorsal view | 0.955 | 0.972 | 0.747 | No |  |  |  |
| Length of ovipositor valves | 0.312 | 0.420 | 0.134 | No |  |  |  |
| Length of first metasomal tergum | 0.035 | 0.166 | 0.0741 | Yes | 0.429 | 0.810 | 0.25 |
| Length of mesopleura | 0.057 | 0.201 | 0.0174 | Yes | 0.0934 | 0.0934 | 0.0101 |
| Length of the eye in frontal view | 0.122 | 0.119 | 0.735 | No |  |  |  |
| Length of the marginal vein | 0.619 | 0.716 | 0.702 | No |  |  |  |
| Length of the stigmal vein | 0.698 | 0.845 | 0.717 | No |  |  |  |

**Table S14.** P-values from Shapiro-Wilk and Kolmogórov-Smirnov normality tests and P-values from Levene's homoscedasticity test according to populations from Santa Cruz Island. The variables transformed by Box-Cox are also presented.

| **Test for slope differences (P)** | LR (likelihood ratio) | p | b | ci | varb | lambda | df (degrees of freedom) |
| --- | --- | --- | --- | --- | --- | --- | --- |
| Mesoscutum length in dorsal view | 0.068 | 0.793 | 1.343 | 1.189 1.516 | 0.006 | 1.806 | 1 |
| Mesoscutum width in dorsal view | 0.159 | 0.689 | 0.987 | 0.878  1.113 | 0.003 | 0.976 | 1 |
| Eye length in dorsal view | 6.867 | 0.008 | 1.082 | 0.924  1.276 | 0.007 | 1.172 | 1 |
| Ovipositor valves length | 1.170 | 0.279 | 1.205 | 1.089  1.329 | 0.003 | 1.453 | 1 |
| First metasomal tergum length | 2.955 | 0.085 | 1.689 | 1.434  2.000 | 0.019 | 2.855 | 1 |
| Mesopleura length | 0.140 | 0.707 | 1.039 | 0.934  1.158 | 0.003 | 1.080 | 1 |
| Eye length in frontal view | 3.245 | 0.071 | 1.520 | 1.273  1.823 | 0.018 | 2.312 | 1 |
| Marginal vein length | 1.642 | 0.200 | 0.976 | 0.870  1.091 | 0.003 | 0.953 | 1 |
| Stigmal vein length | 0.422 | 0.515 | 1.060 | 0.925 1.218 | 0.005 | 1.125 | 1 |

**Table S15.** Statistics of tests for slope differences in allometric relationships. Mesotibia length is the size descriptor.

| **Test for height differences (P)** | stat | p | a | ci | df |
| --- | --- | --- | --- | --- | --- |
| Mesoscutum length in dorsal view | 0.744 | 0.388 | 0.043 | 0.028  0.058 | 1 |
| Mesoscutum width in dorsal view | 0.643 | 0.422 | -0.061 | -0.071  -0.050 | 1 |
| Eye length in dorsal view | 4.332 | 0.037 | -0.199 | -0.214  -0.185 | 1 |
| Ovipositor valves length | 13.622 | 0.0002 | 0.026 | 0.015  0.036 | 1 |
| First metasomal tergum length | 0.005 | 0.938 | -0.331 | -0.356  -0.306 | 1 |
| Mesopleura length | 7.404 | 0.006 | 0.127 | 0.117  0.137 | 1 |
| Eye length in frontal view | 0.283 | 0.594 | -0.228 | -0.252  -0.204 | 1 |
| Marginal vein length | 3.470 | 0.062 | 0.082 | 0.073  0.092 | 1 |
| Stigmal vein length | 34.380 | 4.531e-09 | -0.678 | -0.691  -0.665 | 1 |

**Table S16.** Statistics of tests for height differences in allometric relationships. Mesotibia length is the size descriptor.
